# Miscellaneous

**Published:** 1892-10

**Authors:** 


### MISCELLANEOUS.

### UNWHOLESOME EGGS.

The character of a hen's egg is something that affects consumers of this kind of food very seriously. Few persons suspect danger existing in an egg. There is an old adage to the effect that an egg and a nut can be eaten without suspicion, but it is very far from being true. For a nut has almost always a worm hiding in the kernel, and an egg has been found to have the germs of various loathsome kinds of organisms existing within its substance. This fact has been heretofore mentioned as derived from personal experience of the writer, and now we have before us a report of investigation by Dr. Gayon, who has discovered in eggs bacteria, aspergilli, and other organisms which are derived from the fowl itself, and are to be found also in ovaries and oviduct and blood of diseased fowls. Inoculation of the hens with bacilla resulted in the presence of these organisms in the eggs, and the fecundated eggs were found to be far more profusely supplied than the sterile ones. Consequently even eggs are to be eaten with fear and trembling and the long-boiled hard egg will be far safer than the light-boiled soft ones, and the well cooked omelette safer than either. The owners of fowls should therefore be especially careful of the health of their flock.

The flesh of diseased animals is very properly objected to as food. But the egg of a diseased hen is as much diseased as the flesh. Poultry cholera, roup and other virulent diseases are more prevalent in fowls than any diseases in other animals. Almost every farm flock has its receptacle for departed sick fowls back of the barn or in a fence corner, and in little graves in the garden under the currant bushes or grape vines. No notice is taken of the fact that the eggs of these hens have been gathered and sold or used for weeks preceding the final event, or a thought given that they were virulently unwholesome. Yet we have been told that hens had received the germs of diphtheria (which is roup in their case) and of tuberculosis from human subjects. But who has seriously considered the danger of infection by diphtheria or consumption, or of intestinal fever (which is the foul cholera) from the eggs we eat. And yet there is imminent danger of it that has been heretofore unannounced, so far as we know, except for some years past by the writer in these columns and by Dr. Gayon.

### WHAT PASSES FOR BEAUTY.

The ladies of Arabia stain their fingers and toes red and their lips blue. In Persia they paint a black streak around their eyes and ornament their faces with representations of various figures.

The Japanese women adopt the singular method of gilding their teeth, and those of the Indians paint them red. In some parts of India the pearl of the tooth must be dyed black before a woman can be beautiful. The Hottentot women paint the entire body in compartments of red and black. In Greenland the women color their faces with blue and yellow, and frequently tattoo their bodies by saturating threads in soot, inserting them beneath the skin and then drawing them through.

In New Holland the women cut themselves with shells, and, keeping the wounds open a long time, form deep scars in the flesh, which they deem highly ornamental. Another singular mutilation is made among them, for when in infancy they take off the little finger of the left hand at the second joint. In ancient Persia an aquiline nose was often thought worthy of the crown, but the Sumatran mother carefully flattens the nose of her daughter.

The modern Persians have a strong aversion to red hair. The Turks, on the contrary, are warm admirers of it. In China, small, round eyes are liked. But the great beauty of a Chinese lady is in her feet.

An African beauty must have small eyes, thick lips, a large flat nose and a skin beautifully black. In New Guinea the nose is perforated and a large piece of wood or bone inserted. On the northwest coast of Africa an incision more than two inches long is made in the lower lip an dthen filled with a wooden plug.

American women compress their figures into queer shapes! Well, we all know how it's done!—*Jenness Miller's Monthly.*

---

### COINS WITH QUEER STORIES.

Numismatists, or coin collectors, have interesting objects of search in two coins which belong to the transition period between the French republic and the second empire. One of these is an extremely rare coin which was struck off just at the moment of the assumption of the reins of empire by Napoleon III. Only the die for the obverse or head of a new imperial coin had been completed, and by some accident, or possibly by mischievous design, a coin was struck off which bore the head of "Napoleon III., Emperor," on one side, and "French Republic," on the other.

This contradictory coin is of interest to others that numismatists, for it symbolizes in a striking way the many sudden changes which have taken place in French politics in the past century.

With the other coin a singular story is connected. While Louis Napoleon was "prince-president," and just before he made himself emperor, a decree was issued ordering a five-franc piece to be coined bearing his image.

The dies were made, and the coin was struck off as a sample and sent to the prince-president for approval. But some time passed before he examined it. When at last he gave it his attention he was annoyed to find that he had been represented on the coin with a "love lock" or hooked lock of hair on the temple, which he did actually wear at that period, but which he thought unsuitable to so dignified and permanent a representation of himself as an effigy upon a coin.

The prince-president sent for the director of the mint and ordered him to remove the "love locks." Then he found that his silence with regard to the piece had been taken for approval, and that the stamping of the coins had commenced.

The work was stopped and the image deprived of its undignified look, but the twenty-three coins that had already been struck off were not destroyed, and are now regarded as of great value.

---

HORSEFLESH AS FOOD.—Horseflesh for food has increased wonderfully in popularity in France. At Paris, the first horse butchery was opened on July 9, 1866, and in that year 902 horses were slaughtered. Through seventeen years the business steadily increased, and the count shows that 203,537 solipeds were consumed in the city. On January 1, 1889, the horse butcheries numbered 132. In other cities of France the output of the horse butcheries is enormous.

Hippophagy is also in great favor at Rotterdam. Horse meat is used there as human food to an extent that is unknown in Denmark, Sweden and Switzerland, as well as in parts of Italy. It is extensively used in Milan, while it is scorned in Turin. In the latter city only fifty-five horses were slaughtered in 1888, and the flesh was used exclusively for feeding the animals of a managerie. A Spanish writer regrets that hippophagy is not adopted in Spain, where it would benefit numerous poor laborers, to whom ordinary meat is an article of luxury on account of its high price. In Paris the price of horse meat is about half that of beef.

France and Depopulation.—It seems probable that the process of depopulation which has fairly commenced in France, where deaths have already exceeded the births by 40,000 in a single year, will prove the cause of unparalleled progress in sanitation and preventive medicine. The unique situation is here presented of the diminution of the death rate having become a question of life or death to the nation. An exchange informs us that at the recent meeting of the new Society for the Protection of Children, Dr. Rochard stated that 250,000 infants die yearly, of whom at least 100,000 could be saved by intelligent care. Stringent laws have been already passed to aid in preventing this great loss of life. It is now illegal for any person to give children under one year of age any solid food except on medical advice, and nurses are forbidden to use nursing bottles having rubber tubes. Efforts are being made also to induce Parisian mothers to nurse their own infants.

The World's Largest Bells.—The largest bell in France will be hung in the beautiful church of the Sacred Heart, which is being completed on the hill of Montmartre, near Paris. The bell, which is the gift of the faithful in the Department of Savoy, weighs about 55,000 pounds. It is ten feet high, with a diameter of about ten feet at the base. Two men could stand inside of it easily. While by no means the largest bell in the world, this big fellow is considerably larger than any other bell in France. The largest bell in the cathedral of Notre Dame weighs less than 40,000 pounds, while that in the famous cathedral of Rheims weighs hardly more than 30,000 pounds.

All these bells sink into insignificance, however, when compared with the great bell at Moscow, which weighs about 500,000 pounds. Next in weight are the bells of Protzkoy, 350,000 pounds; of Pekin, 125,000 pounds; of St. Ivan, in Moscow, 115,000 pounds; of Nankin, 50,000 pounds; of Lisbon, 45,000 pounds, while the great bell at St. Peter's in Rome, weighs 40,000 pounds.

Bow-Legged Children.—Mothers cannot be too strongly warned of the evil of allowing children to walk too young. The bones in a young child's leg are soft, half-cartilaginous, and easily bent. People who urge children to walk prematurely are often responsible for lasting injury. Long before soft bones ought to have any strain put upon them, one sees these poor infants made to stand or walk, and by the time they are two years old, their legs are so bent that they have to be put in irons. One very often sees sad and permanent deformities produced in this way. When children are a year old they should be encouraged to creep, but not to walk till after eighteen months.

IMPORTANT IF TRUE.—It is said that Professors Northnagel and Kahler, of Hamburg, have discovered a potent remedy against cholera. The treatment is neither more nor less than administering to the patients enemas of salt warm water. It is claimed by those who have followed this course of treatment that its result is marvelous. In some cases where the patients were in such a state of collapse that it was impossible to discern the pulse, recovery has followed the application of the enemas. All which is highly important, if true.

HOT WATER FOR SPRAINS.—Hot water is the best thing that can be used to heal a sprain or bruise. The wounded part should be placed in water as hot as can be borne for fifteen or twenty minutes, and in all ordinary cases the pain will gradually disappear. Hot water applied by means of cloths is a sovereign remedy for neuralgia or pleurisy pains. For burns or scalds apply cloths well saturated with cool alum water, keeping the injured parts covered from the air.

GREASE SPOTS can be taken out of white marble by an application of whiting saturated with benzine. Let the mixture stand a short time, and then wash off with soap and water. Another mixture to be used in the same way consists of two parts of washing soda and one part each ground pumice-stone and chalk, all finely powdered and mixed into a paste with water.

FOR A BURN.—Cover a burn with vaseline, putting it on thickly. Over this dust corn starch. If you have no vaseline use linseed oil or sweet oil or warm mutton tallow ; and if you have no corn starch use wheat flour. Wrap in a soft linen or cotton cloth.

IT frequently happens that painters splash the plate or other glass windows when they are painting the sash. When such is the case, melt some soda in very hot water and wash them with it, using a soft flannel. It will entirely remove the paint.

